# Environmental (Saprozoic) Pathogens of Engineered Water Systems: Understanding Their Ecology for Risk Assessment and Management

**DOI:** 10.3390/pathogens4020390

**Published:** 2015-06-19

**Authors:** Nicholas J. Ashbolt

**Affiliations:** School of Public Health, University of Alberta, Rm 3-57D South Academic Building, Edmonton, AB T6G 2G7, Canada; E-Mail: ashbolt@ualberta.ca; Tel.: +1-780-492-5227; Fax: +1-780-492-9070

**Keywords:** *Legionella*, *Mycobacterium avium* complex, biofilms, opportunistic pathogens, drinking water, sapronoses, amoeba, *Acanthamoeba*, VBNC, QMRA

## Abstract

Major waterborne (enteric) pathogens are relatively well understood and treatment controls are effective when well managed. However, water-based, saprozoic pathogens that grow within engineered water systems (primarily within biofilms/sediments) cannot be controlled by water treatment alone prior to entry into water distribution and other engineered water systems. Growth within biofilms or as in the case of *Legionella pneumophila*, primarily within free-living protozoa feeding on biofilms, results from competitive advantage. Meaning, to understand how to manage water-based pathogen diseases (a sub-set of saprozoses) we need to understand the microbial ecology of biofilms; with key factors including biofilm bacterial diversity that influence amoebae hosts and members antagonistic to water-based pathogens, along with impacts from biofilm substratum, water temperature, flow conditions and disinfectant residual—all control variables. Major saprozoic pathogens covering viruses, bacteria, fungi and free-living protozoa are listed, yet today most of the recognized health burden from drinking waters is driven by legionellae, non-tuberculous mycobacteria (NTM) and, to a lesser extent, *Pseudomonas aeruginosa*. In developing best management practices for engineered water systems based on hazard analysis critical control point (HACCP) or water safety plan (WSP) approaches, multi-factor control strategies, based on quantitative microbial risk assessments need to be developed, to reduce disease from largely opportunistic, water-based pathogens.

## 1. Introduction

John Snow was possibly the first to ascribe cholera as a drinking water disease in modern times [1] (noting first description of the agent by Pacini [2]), and the subsequent work of Robert Koch cemented the germ theory of disease [3]. This led to the very successful approach to control waterborne (enteric) diseases via centralized drinking water and sanitation treatment systems [4]. However, managing faecally-polluted source waters by way of drinking water treatment has both blinkered our view to and previously overshadowed the broader suite of environmental pathogens potentially present in engineered water systems. In addition to the classic faecal-water-oral route of transmission by waterborne pathogens, there are many more environmental, often opportunistic pathogens, which may colonize drinking water distribution systems (DWDSs) and the plumbing of buildings/homes (premise plumbing) [5]. For the purpose of this review, these systems are referred to as engineered water systems.

This review describes recent information on non-enteric, environmental (saprozoic) pathogens and their primary growth niche, the biofilms that form on pipe surfaces and sediment within engineered water systems; noting recent reviews in this field [6,7,8]. Furthermore this paper takes a risk assessment perspective intended to assist with potential management options—of particular value for hazard analysis critical control point (HACCP) or water safety plans like those recently drafted by the American Society of Heating, Refrigerating and Air-Conditioning Engineers, Inc. (1791 Tullie Circle, N.E. Atlanta, GA, USA) [9] and recommended by the World Health Organization [10].

## 2. Non-Enteric Environmental (Saprozoic) Pathogens (that Cause Sapronoses)

As described by Hubálek [11], sapronoses (Greek “sapros” = decaying; “sapron” means in ecology a decaying organic substrate) are human diseases transmissible from largely abiotic environments (soil, water, decaying plants, or from development within animal corpses, excreta, and other substrata). In essence from plant, invertebrate, and microbial matter—hence saprozoic pathogens include microorganisms in biofilms [12] of engineered water systems [5]. Given their fundamentally different origins compared to enteric pathogens, the generic term “waterborne” originally described for enteric pathogen contamination of water (faecal-oral route) is not appropriate for saprozoic pathogens, rather the general term water-based is preferred; Indicating their water origin, which may also enhance recognition for different control measures (*i.e.* post centralized water treatment). A good example that highlights the need for this differentiation is seen from the current U.S. Environmental Protect Agency (EPA) safe drinking water act (SDWA). In the SDWA there is a goal to have zero *Legionella* within the DWDS, assumed to be possible, as for enteric pathogens via traditional drinking water treatment [13]. However, various legionellae may not only break through treatment within amoeba cysts and from growth in water [14], but enter post treatment, such as via dust into storage tanks where, unlike enteric pathogens, they may grow to high densities [15] and seed downstream biofilms. Depending on premise plumbing conditions, they may then grow to densities likely to impact on unintended bystanders and customer health [16].

Example sapronoses include various diseases possibly caused by amoebal viruses (e.g., *Acanthamoeba polyphaga* mimivirus pneumonia), bacteria (e.g., legionellosis, folliculitis), fungi (e.g., aspergillosis, candidiasis), and free-living protozoan (e.g., primary amoebic meningoencephalitis) [5]. However, obligate intracellular parasites of living (non-human) vertebrates (viruses, rickettsiae, and some chlamydiae) are not considered saprozoic, but rather “sapro-zoonoses” given vertebrate and environmental development sites [11]. And it gets more complicated with some pathogens, such as *Campylobacter jejuni* and *Escherichia coli* O157:H7 that are common to the gastrointestinal tract of vertebrates (*i.e.*, enteric), but may also have natural reservoirs within environmental amoebae [17]; and others enterics like *Acinetobacter baumannii* and *Aeromonas hydrophila* that also grow in engineered water systems (*i.e*., these are all facultative saprozoic). In general, it can be assumed that saprozoic pathogens have evolved over millennia to grow in various environmental niches, and that human infection is largely accidental or a dead-end life-stage. For example, lung infections by *Legionella pneumophila* or various non-tuberculous mycobacteria (NTM) are almost exclusively not spread person-to-person [18,19], presumably ending the pathogens’ continued development. With increasing global climate impacts and demographic change, as with zoonoses, it is highly likely that there will be greater potential for new sapronoses, requiring a one-health approach to management [20]. It is therefore of interest to identify possible generic measures so as to minimize future sapronoses via water systems. Table 1 provides a listing of sapronoses relevant to engineered water systems (modified from [5]), and a sub-set are expanded upon in the follow subsections.

### 2.1. The Key Niche, Biofilms in Engineered Water Systems

Common to moist surfaces is the development of a conditioning film of chemicals that rapidly leads to the development of bacterial biofilms [21], which subsequently support a range of free-living protozoa, metazoan, and other invertebrates in engineered water systems [21,22,23,24,25,26,27,28,29]. Given this ubiquitous growth of biofilms on moist surfaces, it seems fruitless to try to eliminate biofilm development simply by way of maintaining a residual disinfectant in drinking water systems. Rather, specific members may be controlled, such as trophozoites of the free-living protozoan pathogen *Naegleria fowleri* by a chlorine residual [30], or culturable (yet not necessarily all active cells [see VBNC below]) of *L. pneumophila* by monochloramine or copper/silver ions [31]. While biofilm heterotroph growth is generally governed by availability of organic carbon [32], yet sometimes N or P may be limiting [22,33,34,35], there is a complex interaction between residual disinfectant, pipe materials, and hydraulic regime influencing the microbial diversity of biofilms within engineered water systems, as discussed next. Also, upstream biofilm members appear to influence the composition of downstream biofilms [36], and certain phenotypic features of bacteria, such as the presence of fimbriae and autoaggregation influence biofilm formation by individual members [37,38,39]. Autoaggregation by intracellular pathogens like *L. pneumophila* may also increase their uptake by biofilm amoebae [40], leading to a higher likelihood of selecting for virulent biofilm community members [41].

Various approaches have been developed to study biofilms in engineered water systems [12,42,43,44], and what is clear is that both the type of disinfectant and residual concentration appear to influence biofilm microbial diversity, but not eliminate it [33,45,46,47]. A further influencing factor of biofilm community structure is the substratum (e.g. pipe material); for example, with generally greater biofilm growth on plastic pipes compared to copper, which, despite less biofilm copper may support members more likely to increase legionellae presence [48,49,50]. While expected, only recently have studies identified a further nuance of engineered water system biofilms, temporal dynamics of biofilm members due to changes in hydraulics and water chemistries [51,52]. Once transcriptomics become more readily available to study environmental biofilms, further dynamics in expression of functional genes will be evident (e.g. [53,54,55].

**Table 1:** Potential Sapronoses from Pathogens in Engineered Water Systems.

Microbial Group	Agent		Problematic Niche	Sapronose	Ref.
Bacteria	Acinetobacter baumannii*		Free-living within biofilms of health-care settings	Range of nosocomial respiratory & other infections (via biofilms) from drinking water, breathing tubes & urinary catheters; antimicrobial resistant strains.	[56]
	Aeromonas hydrophila*		Ubiquitous in aquatic environments, colonize engineered water systems	Most strains do not appear to be of health concern (including enteric members), but some biofilm colonizers may cause wound infections	[57,58,59]
	*Chlamydiales*: *Neochlamydia* spp., *Parachlamydia* spp., *Simkania negevensis*, *Waddlia chondrophila*		Obligate amoeba-resisting bacteria of environmental biofilms	Community acquired pneumonia Abortions in humans (and bovines)	[60,61,62,63]
	*Legionella longbeacheae* *L. micdadei* *L. pneumophila*		Free-living within biofilms, but important pathogens within biofilm amoebae & other protozoa	Legionellosis (from mild Pontiac Fever to severe Legionnaires’ Disease); Community acquired pneumonia	[15,64,65,66]
	Non-tuberculous mycobacteria (NTM); (1) rapid-growers: *Mycobacterium abscessus,* *M. chelonae,* *M. fortuitum*; (2) slow-growers: *M. avium* complex (MAC), *M. ulcerans*		Free-living within biofilms, some appear facultative within biofilm amoebae and other protozoa	Community acquired pneumonia Lymphadenopathy, skin and soft tissue infection	[19,66,67,68]
	*Pseudomonas aeruginosa*		Ubiquitous in aquatic environments, colonize engineered water systems	Folliculitis from pools/spars and various nosocomial infections from plumbing biofilms	[8,69,70,71]
	*Stenotrophomonas maltophilia*		Ubiquitous in aquatic environments, colonize engineered water systems	Range of nosocomial respiratory & other infections (via biofilms) in drinking water, breathing tubes & urinary catheters; antimicrobial resistant strains	[72]
Fungi	*Aspergillus fumigatus* *A. terreus*			Aspergillosis	[73,74]
	*Candida albicans* *C. parapsilosis*			Candidiasis	[75]
	*Exophiala dermatitidis*			Dermatitidis	[75]
Protozoa	*Acanthamoeba* spp.	Many strains appear to only grow saprophyticly; ubiquitous to aquatic biofilm environments		Granulomatous amoebic encephalitis; keratitis; lung & skin infections	[30,76,77,78,79]
	*Balamuthia mandrillaris*	Relatively rare but present in source and treated waters of temperate regions		Granulomatous amoebic encephalitis; lung & skin infections	
	*Hartmonella* spp. *Vahlkampfia* spp.	Many strains appear to only grow saprophyticly; ubiquitous to aquatic biofilm environments		Keratitis	
	*Naegleria fowleri*	Relatively rare but present in source and treated waters over 28 _°_C if inadequate residual disinfectant		Primary amoebic meningoencephalitis	
Viruses	*Mimivirus* (Shan virus) *Mamavirus*	Potentially in various biofilm amoebae, first described in *A. polyphaga*		Weak pneumonia?	[80,81,82]

*Facultative saprozoic.

A particular feature of many biofilm bacteria is the formation of various dormant states, yet the existence of viable (active) but non-culturable cells (VBNC) have been debated for over 30 years [83,84]. However, only recently have VBNC cells been explored by molecular tools within engineered water system biofilms [85]. A particular relevant point for environmental pathogens, and illustrated here for *L. pneumophila*, is that VBNC cells may remain infectious and can be resuscitated within amoebae/lung macrophages [86,87] even after 30–60 min at 70 °C [86]. Indeed, if VBNC forms are part of the normal cell lifecycle, particular care is needed in assaying the efficacy of disinfection systems, *i.e.*, not just relying on agar plate culture-based methods [88,89,90,91].

Given the enhanced cell densities and proximity of different biofilm members, along with stressors such as metal pipes and residual drinking water disinfectants, biofilms within engineered water systems are likely to be a hot-spot for antimicrobial resistance transfer [92] and on-going evolution of sparozoic viral pathogens associated with gene exchange within biofilm amoebae (e.g. [93]). Hence, overall there is a need to develop higher-resolution molecular knowledge to enhance our ability to model and identify risk periods that need to be managed within engineered water systems [52].

#### 2.1.1. Legionellae, Non-Tuberculous Mycobacteria (NTM) and *Pseudomonas aeruginosa*

The documented cases of severe pneumonia (Legionnaires’ Disease) to the less reported milder illnesses associated with Pontiac Fever [94], as with various wound and respiratory non-tuberculous mycobacteria (NTM) infections [68], are likely to be severely underreported. For both of these pathogen groups, water exposures are thought to be the only pathway of concern, whereas person-to-person spread dominates for enteric pathogens. Nonetheless, hospitalization insurance claims from legionellae and NTM infections in the U.S. far exceed all other identified drinking water pathogens [95]. The next most important water-based pathogen identified was *P. aeruginosa*, mainly due to *otitis media*, which may have a water-association, or the less-impacting but clearly water-related folliculitis [69]. Of increasing recognition, however, are nosocomial issues with multi-drug resistant *P. aeruginosa* from healthcare water systems [8]. The focus of this sub-section is on *L. pneumophila* and NTM characteristics relevant to engineered water systems.

Most clinical studies still utilize culture-based methods, so as to obtain isolates for molecular characterization/“fingerprinting” and drug susceptibility testing. However, for environmental studies, quantitative polymerase chain reaction (qPCR) methods are clearly superior in obtaining positive detects for legionellae (72% by qPCR vs culture’s 34% from a review of papers over the last ten years) [96], with similar findings for NTM [97]. In part, this loss of culturability may be related to the formation of a cyst-like state of the infectious form of *L. pneumophila* [98], or simply the slower growth rate/poorer competitiveness on artificial media of target cells [99]. Resuscitation by co-culture growth within amoebae may assist in identifying infectious cells from the environment and allow subsequent cell typing, but is not particularly reliable. Subsequent questions then arise as to how to interpret positive qPCR, given dead and alive cells being detected, and that pre-treatment with propidium monoazide (PMA) or similar reagents before qPCR to identify cell-membrane damage is not always conclusive [90,100].

A critical realization is that while various legionellae may grow freely within engineered biofilms, strains that grow within free-living amoebae appear to have enhanced pathogenicity [18] and would allow for rapid development of the high cell densities thought necessary for infections via aerosolized water [16]. Other natural hosts for *L. pneumophila* may also include ciliates [101] and nematodes [102]. While NTM may also grow in association with various types of amoebae [103], they also clearly represent a major fraction of the total biofilm biomass in drinking water systems [45,47,104]. What is much less clear is how to identify the sub-fraction that maybe opportunistic human pathogens. One option has been to just focus on the *Mycobacterium avium* complex (MAC), which are more likely to contain human pathogenic strains [105,106]. Nonetheless, it is unclear what characteristics may identify pathogenic strains within the MAC.

## 3. Risk Assessment and Risk Management of Engineered Water Systems 

### 3.1. Quantitative Microbial Risk Assessment (QMRA)

To identify, prioritize, and aid in the management of hazardous pathogen events within engineered water systems, QMRA is emerging as a useful tool to address enteric pathogens, and is beginning to be used for sapronoses [107,108]. The fundamental steps in undertaking a QMRA are to identify the hazards (pathogens) relevant to your system, characterize human exposures (doses), apply relevant dose-response equations for the estimated doses/pathways, and then characterize the risks [109]. It is also important to document uncertainties and undertake at least some form of sensitivity analysis to identify key QMRA model parameters. As is described above, there are very large uncertainties in estimating concentrations of relevant saprozoic pathogens, not only due to a limited sub-set, if any can be cultured from environmental samples, but that current PCR estimates also include infectious and non-infectious members. Further, there are very limited dose-response studies for *L. pneumophila* [16] and *P. aeruginosa* [69], with no available NTM dose-response equation yet.

Therefore, risk assessments for saprozoic pathogens are probably most useful in comparing options based on estimated exposure concentrations, rather than disease risk estimates. Nonetheless, estimates of *L. pneumophila* densities to cause Legionnaires’ Disease from drinking waters are likely to be very high (some millions of cells per liter) [16], with epidemiologic data indicating lesser numbers related to Pontiac Fever (thousands of cells per liter) [93]. Hence, the general expectation is that several orders of magnitude higher doses are probably required for saprozoic pathogens compared to enteric pathogens via drinking water [110]; with the possible exception of *Naegleria fowleri* [30] and *Helicobacter pylori* (<1 cell per liter) [111] that may accumulate (unclear if any growth) in drinking water biofilms [112].

A further advantage of undertaking QMRAs is that it helps to identify the most critical data gaps for managing sapronoses. For example, it is highly uncertain what the water to aerosol partitioning coefficients are for legionellae [16] *versus* NTM [113,114], and what density of amoebal hosts may be necessary to reach the high legionellae estimates *versus* densities of NTM growing freely within biofilms (and how close to the exposure point) to be released into the bulk water?

### 3.2. Management of Sapronoses from Engineered Water Systems

Management of saprozoic pathogens starts with minimizing biofilm build-up within your engineered water system, which means minimizing bioavailable carbon, nitrogen and phosphorus, as well as eliminating stagnation zones, and for legionellae and *Naegleria fowleri*, maintaining a disinfectant residual and temperature control (below 20 °C or above 55 °C) [30,64,87]. The value of a disinfectant residual for management of NTM is likely to be more problematic, as the fraction of NTM within biofilms appears to increase with a disinfectant residual [45,47]. However, specific data on the selection of pathogenic NTM by a disinfectant residual is still largely absent. What is known is that freely suspended NTM cells may be >600 times more resistant to free chlorine than *E. coli* [115], and amoeba-resisting pathogens like *Simkania negevensis* are also more chlorine resistant than legionellae [63]. Also, VBNC and persister state cells within biofilms mean that once established, there will be recalcitrant contamination by saprozoic pathogens that effectively are not possible to remove without pipe replacement or full sterilization. Hence, many recommend point-of-use filters in healthcare settings and demonstrate efficacy [116,117], yet NTM are well known to also colonize these filters [118] so regular replacement is necessary. 

In summary, there is growing recognition that an integrated biofilm pathogen management approach is probably required for long-term control. This could include minimizing biofilm nutrients, and developing conditions that select for a biofilm microbiome that is actively antagonistic to saprozoic pathogens—a probiotic approach [119]. Furthermore, such a probiotic approach may mean keeping a dynamic system, given the nature of pathogens like *Legionella* to adapt to killing its predators [120]. Monitoring the efficacy of such a control strategy may also rely on keeping watch of keystone community members. For example, increasing concentrations of *Acanthamoeba* spp. and *Vermamoeba vermiformis* trophozoites may signal conditions favorable to explosive growth of *L. pneumophila* [121]*.*

## 4. Conclusions

There is still much to be understood before long-term, resilient management options are more common place to deal with what is probably the highest health burden pathogen group via urban water exposures in developed regions, *i.e.*, water-based pathogens that cause sapronoses. Nonetheless, excellent progress has been made over the last decade in regions where water safety management protocols have specifically focused on key members, such as *Legionella* spp. [9,10,122,123]. Combining an understanding of microbial ecology, efficacy of engineering controls, and molecular monitoring approaches is finally progressing this much-needed field of public health management. 
